# Thank you and farewell after 15 years editing respiratory research

**DOI:** 10.1186/s12931-018-0929-2

**Published:** 2018-11-26

**Authors:** Jan Lotvall, Reynold A. Panettieri

**Affiliations:** 1 University of Gothenburg, Gothenburg, Sweden; 2 Rutgers, The State University of New Jersey, New Brunswick, NJ USA

It is with great pride and a strong feeling of accomplishment, we now transition the editorship of Respiratory Research to our capable and accomplished colleagues Kelan Tantisira and Oliver Schildgen. After having served as Co-Editors for over 15 years, we wish to reflect on the legacy of the journal and heartily thank all those who worked so diligently as associate editors, editorial board members and dedicated reviewers, as well as the BioMedCentral staff.

The initial vision for RR was created by the founding editor Professor Peter J. Barnes who saw an unmet need to bridge basic and clinical respiratory research. He therefore helped start this journal as an exclusively web-based publications, almost unheard off at the time. The journal was created in the year 2000, a time when significant challenges existed in the scientific literary world dominated by printed matter, complicated search processes, and inaccessibility to science for anyone outside of a large academic or commercial institution. The concept of open access was totally novel. As editors, the first years were challenging, and in 2003 only 15 papers were published. The scientific community was not familiar with the fundamental changes that Open Access publishing required, such as authors paying for publishing the work. Initially, the journal had no impact factor. However, when the initial impact factor was issued in 2004 the interest from peers suddenly exploded, and in the following year the number of publications increased ten-fold, and has steadily grown with approximately 200 papers now published by RR per year. In parallel, submissions have increased and are approaching 1,000 per year.

When we assumed the Co-Editorship of RR in the spring of 2003 we embraced the contemporary concept of translational medicine and science that focused on fundamental studies using human cells, tissues or model systems that would translate basic observations into clinical studies. This approach was intrinsically bidirectional in nature; the insights from RR articles could generate novel questions that could be pursued by studies focused on the discovery of molecular mechanisms of disease. Most importantly, the open access of RR offered a global audience the rapid dissemination of scientific knowledge into clinical practice. The Journal boasts of a global focus with a consistent recruitment of international editors, associate editors and reviewers who provide the critical vision to publish cutting-edge, interdisciplinary work.

We challenge the new Co-editors Kelan and Oliver to sustain the RR legacy and wish them the best in propelling RR to new heights. Providing respiratory scientists and clinicians with the highest quality translational research in a timely and easily accessible manner, remains the critical RR mission.

Last, but not least, we wish to thank all the authors of the 2126 papers that have been published in Respiratory Research since we took on the editorship 15 years ago, as we could not have done this without your relentless support. Thank you also to all readers of the journal, who bring progress to science and translate basic science to clinical practice. We are both grateful and humbled for having had the opportunity to harness Respiratory Research for such a significant period of time, and wish the best of luck to Kelan and Oliver!

Reynold Panettieri and Jan Lötvall.

